# Prediction of Ki-67 expression in gastric gastrointestinal stromal tumors using histogram analysis of monochromatic and iodine images derived from spectral CT

**DOI:** 10.1186/s40644-024-00820-6

**Published:** 2024-12-31

**Authors:** Xianwang Liu, Tao Han, Yuzhu Wang, Hong Liu, Juan Deng, Caiqiang Xue, Shenglin Li, Junlin Zhou

**Affiliations:** 1 Department of Radiology, Lanzhou University Second Hospital, Cuiyingmen No.82, Chengguan District, Lanzhou, 730030 People’s Republic of China; 2 Second Clinical School, Lanzhou University, Lanzhou, People’s Republic of China; 3 Key Laboratory of Medical Imaging of Gansu Province, Lanzhou, People’s Republic of China; 4 Gansu International Scientific and Technological Cooperation Base of Medical Imaging Artificial Intelligence, Lanzhou, People’s Republic of China

**Keywords:** Gastrointestinal stromal tumor, Histogram analysis, Ki-67 expression, Spectral, Tomography, X-ray computed

## Abstract

**Purpose:**

To assess and compare the diagnostic efficiency of histogram analysis of monochromatic and iodine images derived from spectral CT in predicting Ki-67 expression in gastric gastrointestinal stromal tumors (gGIST).

**Methods:**

Sixty-five patients with gGIST who underwent spectral CT were divided into a low-level Ki-67 expression group (LEG, Ki-67 < 10%, *n* = 33) and a high-level Ki-67 expression group (HEG, Ki-67 ≥ 10%, *n* = 32). Conventional CT features were extracted and compared. Histogram parameters were extracted from monochromatic and iodine images, respectively. The diagnostic efficiency of the histogram parameters from monochromatic and iodine images was assessed and compared between the two groups. Spearman’s correlation analysis was used to correlate histogram parameters with Ki-67 expression.

**Results:**

The HEG was more likely to present with an irregular shape and a larger size than the LEG (all *p* < 0.05). Regarding histogram parameters, the HEG showed higher maximum, mean, Perc.10, Perc.25, Perc.50, Perc.75, Perc.90, Perc.99, SD, variance, and CV of monochromatic images; higher maximum, Perc.99, and entropy of iodine images, compared with the LEG (all *p* < 0.003125). ROC analysis showed that significant histogram parameters of monochromatic and iodine images allowed for effective differentiation between LEG and HEG. DeLong’s test showed that the diagnostic efficiency of histogram parameters in monochromatic images (Perc.90) was superior to that of iodine images (maximum) (*p* = 0.010). A positive correlation was observed between the significant histogram parameters and Ki-67 expression (all *p* < 0.05).

**Conclusion:**

Both histogram analysis of monochromatic and iodine images derived from spectral CT can predict Ki-67 expression in gGIST, and the diagnostic efficacy of monochromatic images is superior to iodine images.

**Supplementary Information:**

The online version contains supplementary material available at 10.1186/s40644-024-00820-6.

## Introduction

Gastric gastrointestinal stromal tumors (gGIST) are the most common mesenchymal tumors of gastric, accounting for approximately 60% of gastrointestinal stromal tumors. They originate from Cajal cells of the digestive tract or precursor cells of Cajal cells with multi-differentiation potential (mesenchymal stem cells) [1]. gGIST has the potential risk of local recurrence and distant metastasis due to its complex biological behaviors [2, 3]. The malignancy and prognosis of gGIST are closely related to the proliferation of the tumor cells [4–7].

Ki-67 is a nuclear antigen closely related to cell proliferation and can promote the proliferation of tumor cells [4, 5]. As an important biological marker, it quantifies the proliferative activity of tumor cells and is widely applied to evaluate the biological behavior of various tumors [5, 6]. High Ki-67 expression is associated with recurrence, metastasis, and poor prognosis in gGIST patients [4]. Previous studies have proved that Ki-67 expression is also associated with KIT mutation or platelet-derived growth factor receptor alpha (PDGFRA) mutation in patients with gGIST. This association can serve as an important reference for selecting suitable patients for adjuvant administration of imatinib [5]. Ki-67 expression is not only a useful indicator for evaluating tumor heterogeneity and prognosis but also provides an essential reference for the formulation of treatment plans. Therefore, an accurate preoperative assessment of Ki-67 expression is of great clinical significance.

Recently, puncture biopsy has become the primary method for preoperative assessment of Ki-67 expression in gGIST. However, this increases the risk of tumor bleeding, rupture, and metastasis along the needle tract [8]. Furthermore, Ki-67 expression obtained from needle biopsy samples may not fully represent the entire tumor due to tumor heterogeneity, thereby influencing the accuracy of Ki-67 expression assessment [6]. Hence, there is an urgent need to explore non-invasive methods for the comprehensive and accurate preoperative assessment of Ki-67 expression in gGIST.

Spectral CT can generate multiple monochromatic images that offer higher tissue resolution and better visualization of lesion substructure, compared with conventional mixed-chromatic images [9, 10]. Our previous study has demonstrated that a 70 keV monochromatic image is the optimal monochromatic image for evaluating gGIST [10]. In addition, iodine images, a unique type of image derived from spectral CT, visualize the blood supply within the tumor and are quantitatively evaluated by the iodine concentration values [10–12]. Several studies have attempted to evaluate the biological behavior of gGIST using quantitative parameters obtained from 70 keV monochromatic and iodine images [10, 11]. Notably, these studies were limited to local regions of the lesion, and deeper information regarding tumor heterogeneity in 70 keV monochromatic and iodine images was underutilized. Histogram analysis is a non-invasive, quantitative medical image analysis tool that provides a comprehensive assessment of tumor heterogeneity [13, 14].

To the best of our knowledge, no study has evaluated Ki-67 expression in gGIST using histogram analysis of 70 keV monochromatic or iodine images derived from spectral CT. We hypothesized that the histogram analysis of monochromatic and iodine images could assess Ki-67 expression in gGIST. Therefore, this study aimed to assess and compare the diagnostic efficiency of histogram analysis of monochromatic and iodine images derived from spectral CT in predicting Ki-67 expression in gGIST.

## Materials and methods

### Patients

**Fig. 1:**
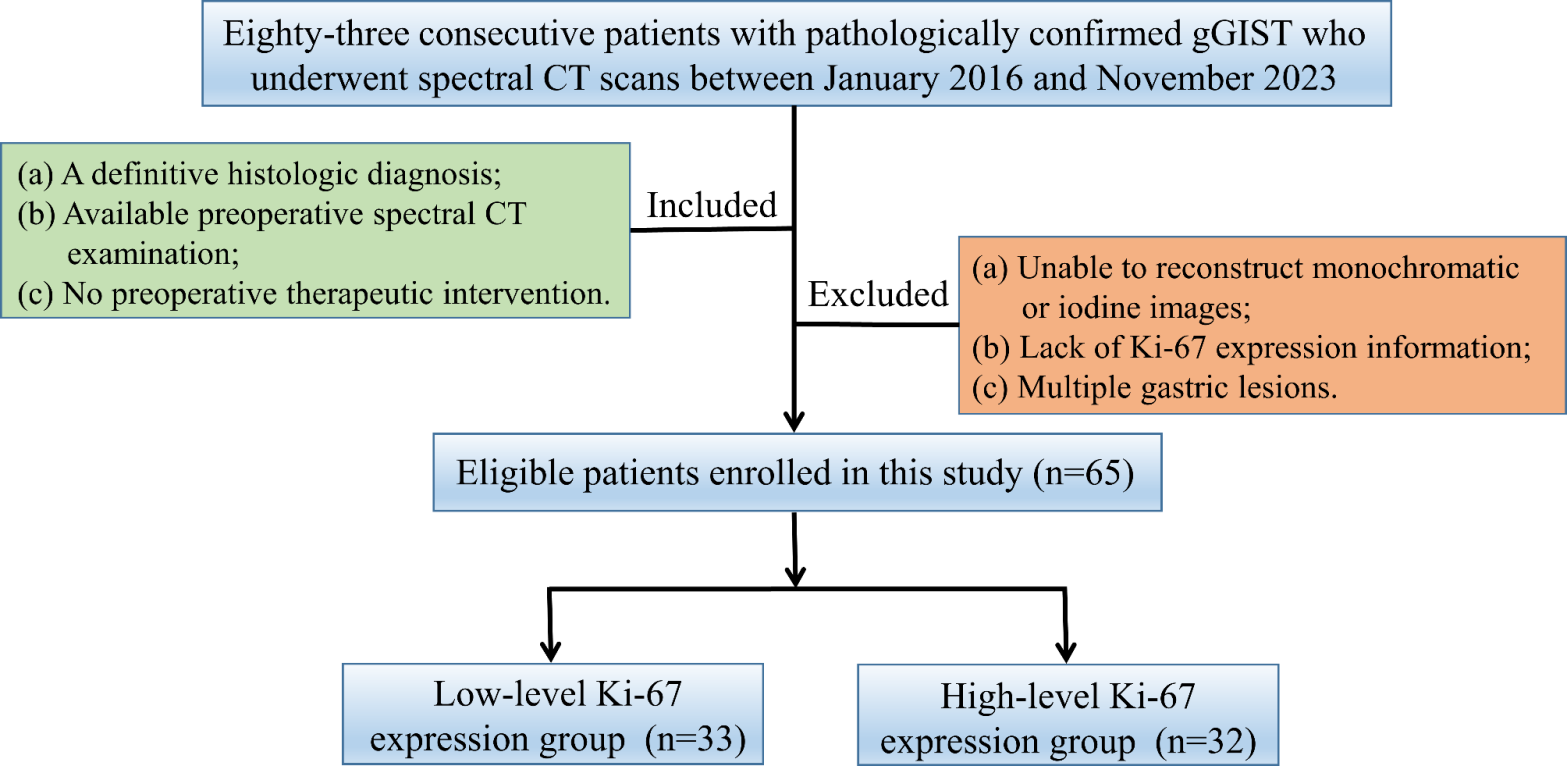
Flowchart shows the inclusion and exclusion processes of patients

This retrospective study was approved by our Institutional Review Board, and the requirement of informed consent was waived. Eighty-three consecutive patients with gGIST who underwent spectral CT between January 2016 and November 2023 were enrolled. The inclusion criteria were: (a) definitive histological diagnosis, (b) availability of preoperative spectral CT examination within one week before the operation, and (c) no preoperative therapeutic intervention. The exclusion criteria were: (a) inability to reconstruct monochromatic or iodine images, (b) lack of Ki-67 expression information, and (c) presence of multiple gastric lesions. Seven patients were excluded due to the inability to reconstruct monochromatic or iodine images, five patients due to lack of Ki-67 expression information, and six patients due to multiple gastric lesions. Finally, 65 patients with gGIST constituted the study cohort. The patient flowchart is presented in Fig. 1.

### CT examination

Spectral CT examinations were performed using the Discovery CT 750 HD (GE Healthcare, Waukesha, WI, USA) and Revolution CT scanner (GE Healthcare, Waukesha, WI, USA). Before the examination, the patient had to fast for 6–8 h and drink 800–1200 ml of warm water to distend the stomach. The scan range was from the top of the diaphragm to the upper edge of the pubic symphysis. A non-enhanced CT scan was performed with the conventional helical scan mode at a tube voltage of 120 kV. Contrast-enhanced CT scan was performed in spectral imaging mode. A non-ionic contrast agent (concentration: 300 mg iodine/mL, iohexol, Ultravist 300, Bayer Pharma) was injected via the elbow vein using a high-pressure syringe (dose: 1.5 mL/kg; rate: 3.0 − 4.0 mL/s) to obtain contrast-enhanced CT images. Arterial, venous, and delayed phase scanning began at the 20s, 80s, and 180s after the trigger attenuation threshold (100 HU) was reached above the level of the abdominal aorta, respectively. The scanning parameters were as follows: rapid switching between tube voltages, 80 kV and 140 kV; tube current, 600 mA; rotation speed, 0.6 s; helical pitch, 0.983:1; collimation thickness, 0.625 mm; slice thickness, 1.25 mm; slice interval, 1.25 mm; and matrix size, 512 × 512. The 70 keV monochromatic and iodine images of the venous phase were reconstructed on an advanced workstation 4.7 (AW 4.7; GE Healthcare), respectively.

### Image analysis

#### Conventional CT features

Two independent, experienced abdominal radiologists (25 and 10 years of experience, respectively) reviewed all images, blinded to the pathological information. Conventional CT features, including location (cardia, gastric fundus, gastric body, or antrum), growth pattern (endophytic, exophytic, or mixed), shape (regular or irregular), margin (clear or unclear), necrosis/cystic changes (yes or no), calcification (yes or no), ulceration (yes or no), intratumoral vessels (yes or no), enhancement pattern (homogeneous or heterogeneous), and size were assessed and recorded [15, 16].

#### Histogram analysis

An open-source FireVoxel software (version: 431 A, https://www.firevoxel.org/) was applied to perform histogram analysis by the two individuals who conducted the qualitative analyses. Firstly, 70 keV monochromatic and iodine images of the venous phase were imported into FireVoxel software on an offline workstation. Then, all slices of the CT images were reviewed, and the slice with the largest tumor area was selected to set the ROI for each patient. The sketched ROIs should exclude the areas of necrosis, cysts, or calcification [17]. Subsequently, the ROI was projected onto iodine images. Finally, histogram parameters (including minimum, maximum, mean, Perc.01, Perc.10, Perc.25, Perc.50, Perc.75, Perc.90, Perc.99, standard deviation (SD), variance, coefficient of variation (CV), skewness, kurtosis, and entropy) were extracted and recorded from monochromatic and iodine images, respectively.

### Ki-67 expression evaluation

All postoperative tumor tissues were paraffin-embedded, sectioned, routinely stained with hematoxylin and eosin, and analyzed for Ki-67 expression using the anti-MIB-1 monoclonal antibody (DakoCytomation, Glostrup, Denmark). Ki-67 was positively expressed in the nucleus as brownish-yellow granules. Ten high-magnification fields under ×400 magnification were randomly selected, 100 cells were counted in each field, and Ki-67 expression was calculated as the number of positive cells divided by the total cell count. According to previous studies, Ki-67 expression < 10% was defined as the low-level Ki-67 expression group of gGIST (LEG), and Ki-67 expression ≥ 10% as the high-level Ki-67 expression group of gGIST (HEG) [17].

### Statistical analysis

Statistical analyses were performed using SPSS software(Version 27.0, Chicago, IL, USA). Categorical variables were compared using the χ2 test, while non-categorical variables were analyzed using an independent *t*-test (for normal distribution) or the Man-Whitney *U* test (for non-normal distribution). Binary logistic regression analysis was used to obtain a combined variable of conventional CT features. Receiver operating characteristic (ROC) curves were conducted to determine the diagnostic efficiency. The Youden index was used to identify the optimal cut-off value from the ROC curve to maximize sensitivity, specificity, accuracy, positive predictive value, and negative predictive value. Spearman’s correlation analysis was used to determine the correlation between the histogram parameters and Ki-67 expression. For the sixteen histogram parameters, Bonferroni corrections were applied for multiple comparisons. Therefore, *p* < 0.003125 (0.05/16) indicated a statistically significant difference. The Delong test was used to compare the differences between areas under the curves (AUCs). Intraclass correlation coefficient (ICC) was used to evaluate the inter-observer reproducibility of the histogram parameters. A *p*-value less than 0.05 was considered statistically significant.

## Results

### Patient clinical characteristics

Of the 65 patients with gGIST in this study, 33 were classified as LEG and 32 as HEG. The detailed clinical characteristics are presented in Table 1. No differences were found in age or sex between the LEG and HEG groups (*p* = 0.936 and *p* = 0.163, respectively).

**Table 1:** Clinical characteristics of gGIST patients in low- and high-level Ki-67 expression group

Characteristics	Ki−67 expression		*P*
	LEG (*n* = 33)	HEG (*n* = 32)	
Age (years)	59.76 ± 8.68	59.97 ± 12.06	0.936
Sex(female/male)	16/17	21/11	0.163
Ki-67 expression (%)	3.73 ± 1.61	17.03 ± 9.28	< 0.001

**Notes**: gGIST, gastric gastrointestinal stromal tumors; LEG, low-level Ki-67 expression group of gGIST; HEG, high-level Ki-67 expression group of gGIST

### Comparison of conventional CT features between low- and high-level expression of Ki-67 in gGIST patients

The conventional CT features of the LEG and HEG are described in Table 2. Compared with the LEG, the HEG was more likely to present with an irregular shape and a larger size (all *p* < 0.05). However, there were no significant differences in location, growth pattern, margin, necrosis/cystic changes, calcification, ulceration, intratumoral vessels, or enhancement pattern between the two groups (*p* = 0.105–0.598).

**Table 2:** Comparison of conventional CT features between low- and high-level expression of Ki-67 in gGIST patients

Parameters	Ki-67 expression		*p*
	LEG (*n* = 33)	HEG (*n* = 32)	
**Location**			0.598
cardia	5	4	
gastric fundus	10	6	
gastric body	16	18	
antrum	2	4	
**Growth pattern**			0.527
endophytic	10	9	
exophytic	20	17	
mixed	3	6	
**Shape**			0.031
regular	25	16	
irregular	8	16	
**Margin**			0.294
clear	29	25	
unclear	4	7	
**Necrosis/cystic changes**			0.504
yes	6	8	
no	27	24	
**Calcification**			0.349
yes	7	4	
no	26	28	
**Ulceration**			0.181
yes	4	8	
no	29	24	
**Intratumoral vessels**			0.174
yes	13	18	
no	20	14	
**Enhancement pattern**			0.105
Homogeneous	19	12	
Heterogeneous	14	20	
**Size (cm)**	3.23 ± 1.80	4.57 ± 1.86	0.004

**Notes**: gGIST, gastric gastrointestinal stromal tumors; LEG, low-level Ki-67 expression group of gGIST; HEG, high-level Ki-67 expression group of gGIST

### Inter-observer reproducibility of histogram parameters

Excellent inter-observer agreement was observed in the histogram parameters of monochromatic (ICCs: 0.857–0.993) and iodine images (ICCs: 0.882–0.995), respectively (Supplementary Material Table S1).

### Comparison of histogram parameters between low- and high-level expression of Ki-67 in gGIST patients

**Fig. 2:**
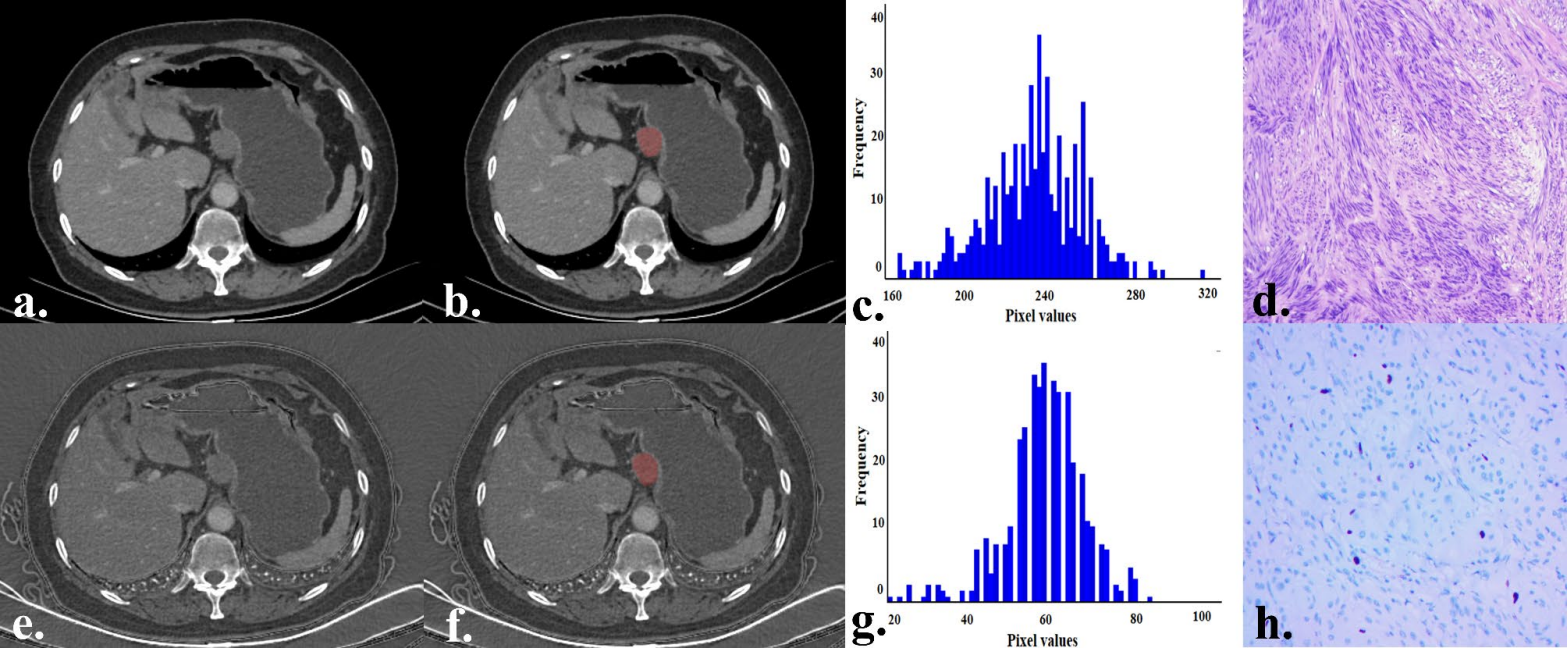
A 59-year-old female with a gGIST belongs to the low-level Ki-67 expression group. The tumor is located in the body of the gastric, has a regular shape, and exhibits a growth pattern of exophytic (**a**,** e**). The tumor ROI was sketched on monochromatic (**b**) and iodine images (**f**). The histogram of the ROI in monochromatic (**c**) and iodine images (**g**). Pathologically confirmed gGIST (hematoxylin and eosin, ×100, **d**). Ki-67 expression was 3% (anti-MIB-1 monoclonal antibody, ×200, **h**) gGIST, gastric gastrointestinal stromal tumors; ROI, region of interest

**Fig. 3:**
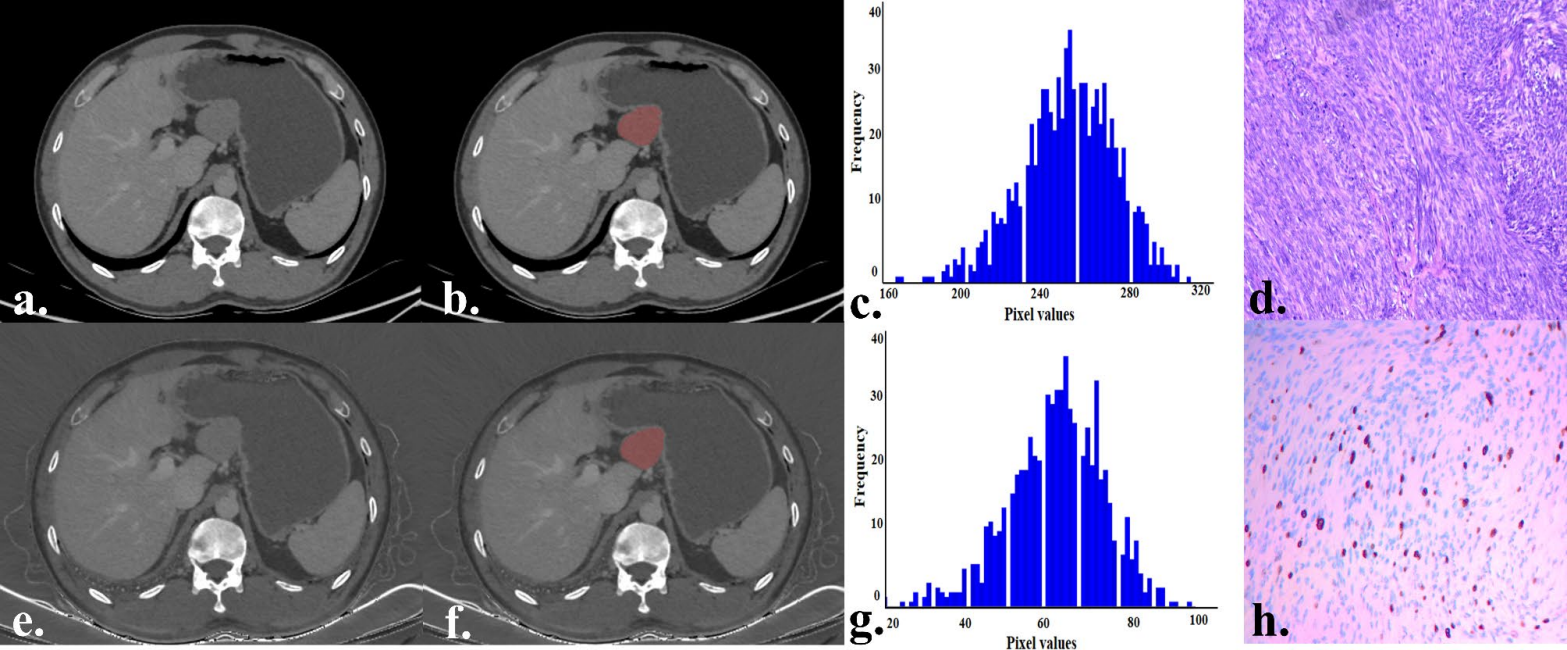
A 40-year-old male with a gGIST belongs to the high-level Ki-67 expression group. The tumor is located in the body of the gastric, has an irregular shape, and exhibits a growth pattern of exophytic (**a**,** e**). The tumor ROI was sketched on monochromatic (**b**) and iodine images (**f**). The histogram of the ROI in monochromatic (**c**) and iodine images (**g**). Pathologically confirmed gGIST (hematoxylin and eosin, ×100, **d**). Ki-67 expression was 15% (anti-MIB-1 monoclonal antibody, ×200, **h**) gGIST, gastric gastrointestinal stromal tumors; ROI, region of interest

Table 3 presents the comparison results of the histogram parameters of the monochromatic and iodine images between the LEG and HEG. In the histogram parameters of monochromatic images, the HEG exhibits high values of maximum, mean, Perc.10, Perc.25, Perc.50, Perc.75, Perc.90, Perc.99, SD, variance, and CV (all *p* < 0.003125). For the histogram parameters of iodine images, the HEG exhibits high values of maximum, mean, Perc.75, Perc.90, Perc.99, SD, variance, CV, skewness, and entropy, compared with the LEG. After Bonferroni correction, only the maximum, Perc.99, and entropy showed a statistically significant difference (all *p* < 0.003125). However, there was no statistical difference in the minimum, Perc.01, skewness, or kurtosis between the two groups (all *p* > 0.003125). Representative cases are shown in Figs. 2 and 3, respectively.

**Table 3:** Comparison of histogram parameters between low- and high-level expression of Ki-67 in gGIST patients

Parameters	Ki−67 expression		*p*
	LEG (*n* = 33)	HEG (*n* = 32)	
Monochromatic images			
Minimum^b^	192.00 (173.50, 213.00)	189.50 (175.25, 208.25)	0.679
Maximum^b^	299.00 (285.00, 314.00)	344.50 (326.00, 366.00)	< 0.001*
Mean^b^	255.00 (248.50, 260.00)	266.00 (262.25, 275.75)	< 0.001*
Perc.01^b^	213.00 (205.50, 222.00)	216.50 (208.50, 229.00)	0.279
Perc.10^b^	235.00 (231.00, 239.00)	242.00 (235.25, 251.00)	0.003*
Perc.25^b^	246.00 (240.00, 249.50)	254.50 (250.00, 264.50)	0.003*
Perc.50^b^	256.00 (250.00, 260.50)	267.00 (264.00, 277.25)	< 0.001*
Perc.75^b^	266.00 (258.50, 272.50)	280.50 (274.00, 289.50)	< 0.001*
Perc.90^b^	273.00 (266.00, 282.50)	294.50 (288.00, 299.75)	< 0.001*
Perc.99^b^	287.00 (277.50, 298.00)	318.50 (305.75, 324.00)	< 0.001*
SD^b^	15.84 (13.30, 17.75)	21.92 (18.49, 23.94)	< 0.001*
Variance^b^	236.82 (176.36, 313.96)	513.13 (341.64, 578.35)	< 0.001*
CV^b^	0.06 (0.05, 0.07)	0.08 (0.07, 0.09)	< 0.001*
Skewness^b^	−0.32 (−0.46,−0.12)	−0.29 (−0.44,−0.20)	0.803
Kurtosis^b^	0.50 (0.17, 1.66)	0.65 (0.26, 1.16)	0.854
Entropy^a^	3.87 ± 1.89	3.88 ± 1.86	0.828
**Iodine images**			
Minimum^a^	45.94 ± 5.62	44.69 ± 7.22	0.437
Maximum^b^	77.00 (73.00,79.50)	83.50 (78.25, 95.75)	< 0.001*
Mean^b^	61.00 (58.50,64.00)	63.00 (60.25, 70.00)	0.045
Perc.01^a^	50.39 ± 4.15	51.25 ± 4.92	0.451
Perc.10^b^	56.00 (54.00, 58.00)	56.50 (54.00, 63.00)	0.303
Perc.25^b^	58.00 (56.00, 61.00)	59.50 (57.00, 65.00)	0.151
Perc.50^b^	61.00 (58.50, 64.00)	62.50 (60.25, 71.00)	0.074
Perc.75^b^	64.00 (62.00, 67.50)	66.00 (63.25, 74.00)	0.041
Perc.90^b^	66.00 (64.00, 71.00)	69.00(66.25, 78.00)	0.017
Perc.99^b^	72.00 (69.00, 76.00)	75.00 (73.25, 84.00)	0.001*
SD^b^	4.67 (4.13, 5.62)	5.48 (4.80, 6.00)	0.022
Variance^b^	21.75 (16.99, 31.41)	29.99 (23.25, 35.98)	0.016
CV^b^	0.08 (0.07, 0.09)	0.08 (0.07, 1.00)	0.033
Skewness^b^	−0.04 (−0.21, 0.12)	0.10 (−0.02, 0.21)	0.006
Kurtosis^b^	0.31 (0.03, 0.50)	0.36 (0.11, 0.82)	0.166
Entropy ^b^	2.64 (2.50, 2.87)	3.05 (2.95, 3.16)	< 0.001*

**Notes**: gGIST, gastric gastrointestinal stromal tumors; LEG, low-level Ki-67 expression group of gGIST; HEG, high-level Ki-67 expression group of gGIST; SD, standard deviation; CV, coefficient of variation; ^a^, Data are expressed as mean ± standard deviation and compared by independent *t*-test; ^b^, Data are expressed as median (lower quartile, upper quartile) and compared by Mann–Whitney *U* test

*p value < 0.003125, signifcant diference after Bonferroni correction for multiple comparisons

### Diagnostic efficacy analysis

**Fig. 4:**
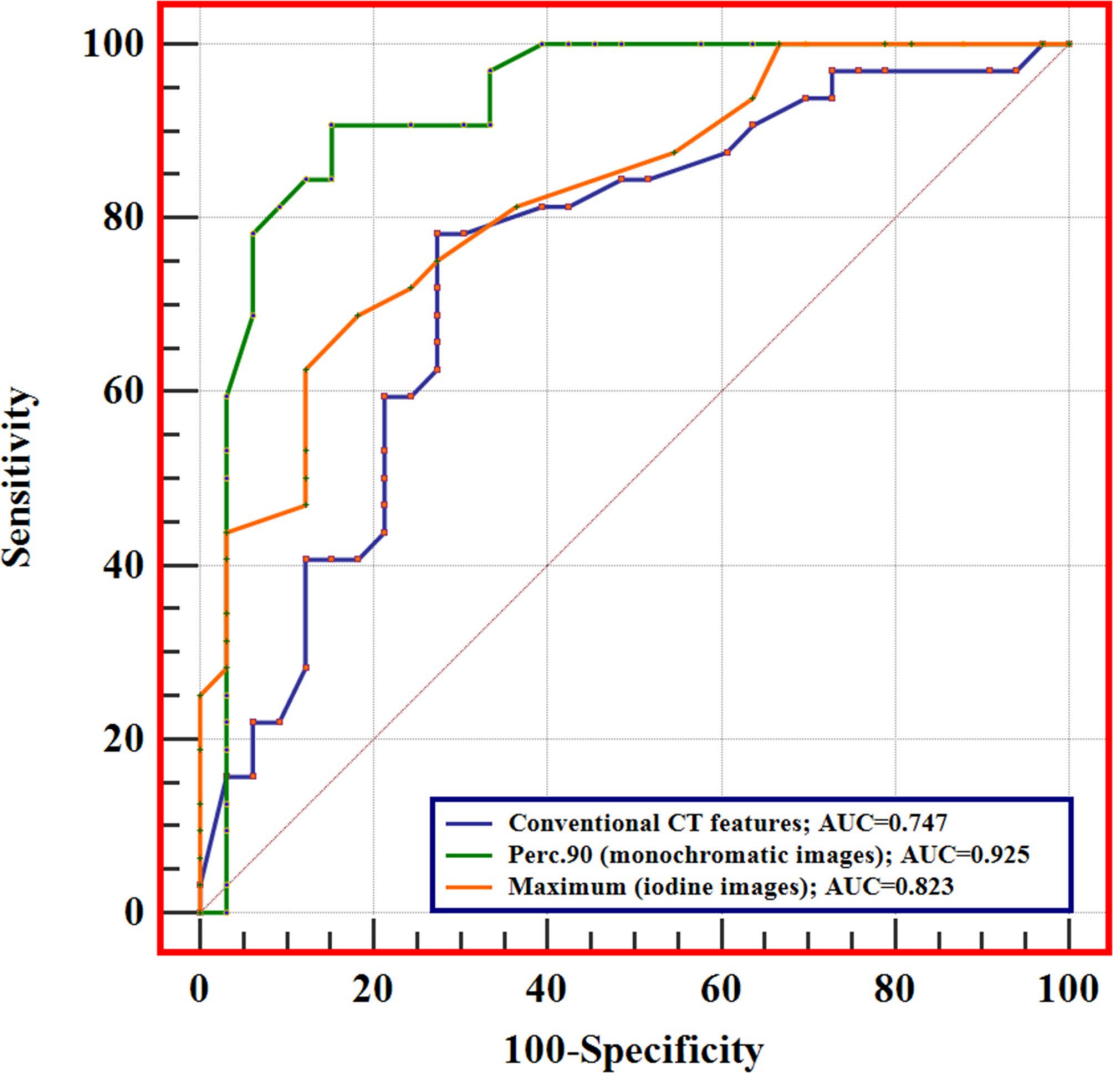
The performance of conventional CT features (combined shape and size), Pec.90 (monochromatic images), and maximum (iodine images), and the diagnostic efficacy of the Pec.90 (monochromatic images) is superior to the maximum (iodine images)

The diagnostic efficacy of conventional CT features and significant histogram parameters of monochromatic and iodine images in distinguishing between LEG and HEG are presented in Table 4. The AUC of conventional CT features (combined shape and size) was 0.747 (95% confidence interval [CI]: 0.624–0.847). In monochromatic images, the Perc.90 generated the highest diagnostic performance (AUC: 0.925, 95% CI: 0.832–0.975), while in iodine images, the maximum exhibited the highest diagnostic performance (AUC: 0.823, 95%CI: 0.709–0.907). DeLong’s test showed that the diagnostic efficiency of histogram parameters in monochromatic images (Perc.90) was superior to that in iodine images (maximum) ( *p* = 0.010, Fig. 4).

**Table 4:** Diagnostic efficacy analysis

Parameters	AUC (95% CI)	Cut-off	Sen (%)	Spe (%)	Acc (%)	Ppv(%)	Npv(%)
**Conventional CT features**							
Shape + Size	0.747(0.624, 0.847)	0.46	78.12	72.73	75.38	73.50	77.40
Maximum	0.875 (0.770, 0.944)	323.00	78.12	90.91	84.62	89.30	81.10
Mean	0.849 (0.739, 0.926)	261.00	78.12	93.94	86.15	92.60	81.60
Perc.10	0.714 (0.588, 0.819)	240.00	53.13	90.91	72.31	85.00	66.70
Perc.25	0.811 (0.695, 0.898)	249.00	81.25	75.76	80.00	76.50	80.60
Perc.50	0.889 (0.786, 0.953)	262.00	87.50	90.91	89.23	90.30	88.20
Perc.75	0.921 (0.827, 0.974)	270.00	100.00	69.70	84.62	76.20	100.00
Perc.90	0.925 (0.832, 0.975)	283.00	90.62	84.85	87.69	85.30	90.30
Perc.99	0.886 (0.783, 0.951)	307.00	75.00	96.97	86.15	96.00	80.00
SD	0.786 (0.667, 0.878)	17.66	84.37	75.76	80.00	71.10	83.30
Variance	0.795 (0.677, 0.885)	309.69	84.37	75.76	80.00	71.10	83.30
CV	0.773 (0.652, 0.868)	0.06	90.62	63.64	78.46	70.70	87.50
**Iodine images**							
Maximum	0.823 (0.709, 0.907)	80.00	68.75	81.82	75.38	78.60	73.00
Perc.99	0.731 (0.607, 0.834)	71.00	96.87	45.45	70.77	63.30	93.70
Entropy	0.794 (0.676, 0.884)	2.87	90.62	78.79	84.61	80.60	89.70

**Notes**: SD, standard deviation; CV, coefficient of variation; AUC, area under the receiver operating characteristic curve; CI, confidence interval; Sen, sensitivity; Spe, specificity; Acc, accuracy; Ppv, positive predictive value; Npv, negative predictive value

### Correlation between significant histogram parameters and the Ki-67 expression

Correlations between the significant histogram parameters of monochromatic and iodine images and Ki-67 expression are shown in Table 5. In the histogram parameters of monochromatic images, positive correlations were observed between the maximum (*r* = 0.649), mean (*r* = 0.615), Perc.10 (*r* = 0.352), Perc.25 (*r* = 0.506), Perc.50 (*r* = 0.674), Perc.75 (*r* = 0.734), Perc.90 (*r* = 0.742), Perc.99 (*r* = 0.756), SD (*r* = 0.555), variance (*r* = 0.580), and CV (*r* = 0.531) and Ki-67 expression (all *p* < 0.05). In the histogram parameters of the iodine images, maximum (*r* = 0.621), Perc.99 (*r* = 0.464), and entropy (*r* = 0.522) showed positive correlations with Ki-67 expression (all *p* < 0.05).

**Table 5:** Correlation between significant histogram parameters and the Ki-67 expression

Parameters	Ki−67 expression	
	*r*	*p*
**Monochromatic images**		
Maximum	0.649	< 0.001
Mean	0.615	< 0.001
Perc.10	0.352	0.004
Perc.25	0.506	< 0.001
Perc.50	0.674	< 0.001
Perc.75	0.734	< 0.001
Perc.90	0.742	< 0.001
Perc.99	0.756	< 0.001
SD	0.555	< 0.001
Variance	0.580	< 0.001
CV	0.531	< 0.001
**Iodine images**		
Maximum	0.621	< 0.001
Perc.99	0.464	< 0.001
Entropy	0.522	< 0.001

**Notes**: gGIST, gastric gastrointestinal stromal tumors; SD, standard deviation; CV, coefficient of variation; *r* = Spearman’s correlation coefficient

## Discussion

This study assessed and compared the diagnostic efficiency of histogram analysis of monochromatic and iodine images derived from spectral CT in predicting Ki-67 expression in gGIST. Our results indicate that both histogram analysis of monochromatic and iodine images can predict Ki-67 expression in gGIST, and the diagnostic efficacy of monochromatic images is superior to iodine images ( *p* = 0.010).

Ki-67 expression is a reliable biomarker for predicting the biological behavior of gGIST. Previous studies have demonstrated that a high level of Ki-67 expression not only predicts poor prognosis but also contributes to the development of individualized gene-targeted therapeutic regimens for patients with gGIST [7]. Several conventional CT features have been proven to correlate with Ki-67 expression in gGIST [5, 15]. In this study, we found that HEG was more prone to display an irregular shape feature, which can be explained by the active proliferation of HEG tumor cells. The proliferation rate of cells in different regions of the tumor varies due to tumor heterogeneity, resulting in the degree of tumor growth in different directions being inconsistent, and ultimately leading to irregular features. Chen XS et al. [15] found that an irregular shape feature was a significant indicator for predicting the Ki-67 expression in gGIST, which aligns with our study. Meanwhile, larger tumor size was more likely to be observed in HEG in this study, which may also be attributed to high-level Ki-67 expression tumors with higher proliferative activity. Active cell proliferation leads to rapid tumor growth and larger size [18].

Spectral CT is a multiparametric, multimodal imaging technique [19]. In addition to conventional CT features, spectral CT also provides a variety of unique images for the analysis of tumor heterogeneity. Monochromatic and iodine images are the two most commonly used types of images in spectral CT studies [19, 20]. Monochromatic images can improve image quality by reducing image noise and increasing the contrast between tissues, whereas iodine images obtained based on iodine-based material decomposition techniques can visualize iodine uptake by tumors, thus indirectly reflecting the microcirculatory status of the tumor tissues [20, 21]. Previously published spectral CT studies of gGIST have mostly been based on focal regions of tumors, neglecting a large amount of important information contained in monochromatic and iodine images, which is closely related to tumor heterogeneity [10, 11]. Histogram analysis provides a new approach for fully investigating deeper information in monochromatic and iodine images derived from spectral CT. Liang G et al. [22] found that texture parameters obtained from monochromatic images could effectively differentiate between benign and malignant solitary pulmonary nodules. Uhrig M et al. [23] showed that iodine image histogram analysis helps monitor the efficacy of targeted therapies in patients with melanoma. These studies demonstrate the utility and reliability of histogram analysis of monochromatic and iodine images in the assessment of tumor biological behavior. However, no study has explored whether the histogram analysis of monochromatic and iodine images can predict Ki-67 expression in gGIST.

In this study, during histogram analysis of monochromatic and iodine images, we observed that the maximum, mean, Perc.10, Perc.25, Perc.50, Perc.75, Perc.90, Perc.99, SD, variance, and CV of the monochromatic images, the maximum, Perc.99, and entropy of the iodine images were higher in the HEG than in the LEG. These parameters correlated positively with Ki-67 expression and provided effective preoperative differentiation between the two groups of tumors. The maximum, mean, and percentile of histogram parameters obtained from contrast-enhanced images reflect the distribution of image pixels and quantitatively indicate tumor blood supply [24]. In our study, both the maximum, mean and percentile of the histogram parameters obtained in monochromatic and iodine images were larger in the HEG than those in the LEG. This may be because Ki-67 expression increases with the degree of proliferative activity of gGIST tumor cells. High Ki-67 expression induces tumor neovasculogenesis, leading to the tumor with a more abundant blood supply, which is manifested by higher maximum, mean, and percentile values [25]. An interesting finding was observed in the results of ROC analysis, the diagnostic efficacy of the high-level percentile in histogram parameters seems to be superior to the low-level percentile. This may result from that the high-level percentile of CT histogram parameters represents the most actively proliferating and well-vascularized regions within the gGIST tissues, leading to the difference of high-level percentile in histogram parameters being more significant between LEG and HEG. Similar results were also presented in a previous study using CT histogram parameters to predict the histological grading of rectal neuroendocrine tumors [14]. Meanwhile, correlation analysis in our study revealed significantly stronger coefficients between high-level percentile histogram parameters and Ki-67 expression compared to low-level percentile. Based on these findings, we propose that the high-level percentile in histogram parameters of monochromatic and iodine images derived from spectral CT may be suitable for the preoperative assessment of Ki-67 expression in gGIST. Certainly, further research is required to validate our hypotheses.

SD, variance, CV, skewness, and entropy are quantitative metrics that reflect the distribution of histogram parameters, indicating the complexity and inhomogeneity of tumor components; larger values denote stronger tumor heterogeneity [24]. In this study, compared to LEG, HEG exhibited higher SD, variance, and CV from monochromatic images, and higher entropy from iodine images, suggesting that the internal components of the tumors in HGM were more complex, which also consistent with greater heterogeneity in tumors with higher Ki-67 expression [4]. Similarly, a positive correlation was also observed between these parameters and Ki-67 expression in gGIST, and each of them could achieve effective preoperative differentiation between the two groups of tumors. This further demonstrates the feasibility of preoperative prediction of Ki-67 expression in gGIST using histogram analysis of monochromatic and iodine images derived from spectral CT.

In our study, we also compared the diagnostic efficiency of histogram analysis using monochromatic and iodine images in predicting the Ki-67 expression in gGIST. We observed that Perc.90 and maximum were the histogram parameters that yielded the highest AUC values in monochromatic and iodine images, respectively. Statistical analysis showed that the diagnostic efficiency of Pec.90 of monochromatic images was significantly superior to the maximum of iodine images, and the difference between the diagnostic performance was statistically significant. Furthermore, our study also observed that for the same type of histogram parameters extracted from monochromatic and iodine images, the diagnostic efficacy of histogram parameters obtained from monochromatic images was superior to that of iodine images. Additionally, correlation coefficients between histogram parameters from monochromatic images and Ki-67 expression were significantly stronger than those from iodine images in correlation analyses. Therefore, we consider the diagnostic efficacy of histogram analysis of monochromatic images to be superior to that of iodine images in predicting the Ki-67 expression of gGIST. The reason for this is hard to explain. A possible explanation lies in the distinct image characteristics in monochromatic and iodine images. Monochromatic images offer advantages such as reduced beam-hardening artifacts and increased tissue contrast, lower image noise, and a higher contrast-to-noise ratio [9]. The advantage of iodine images is the visualization of iodine uptake in tumor tissue, which allows quantitative analysis of tumor blood supply by analyzing iodine uptake in tumor tissue [26]. Higher image quality may be an important reason for the superior diagnostic performance of the histogram parameters of monochromatic images over those of iodine images.

### Limitations

Our study had some limitations. Firstly, selective bias was inevitable because it was a single-center retrospective study. Secondly, due to the lack of relevant follow-up data, the relationship between histogram parameters of monochromatic and iodine images derived from spectral CT and survival time in gGIST has not been investigated. Last but not least, an independent external validation set is needed to validate our results.

## Conclusion

In conclusion, our study shows that histogram analysis of both monochromatic and iodine images derived from spectral CT is helpful for predicting Ki-67 expression in gGIST, and that the diagnostic efficacy of monochromatic images is superior to that of iodine images ( *p* = 0.010). This finding contributes to the formulation of personalized treatment strategies.

## Electronic supplementary material

Below is the link to the electronic supplementary material.

Supplementary Material 1

## Data Availability

No datasets were generated or analysed during the current study.

## Abbreviations

gGIST: Gastric gastrointestinal stromal tumors
HEG: High-level Ki-67 expression group of gGIST
LEG: Low-level Ki-67 expression group of gGIST
SD: Standard deviation
CV: Coefficient of variation
ROC: Receiver operating characteristic curve
AUC: Area under the curve
ROI: Region of interest
CI: Confidence intervals
ICC: Intraclass correlation coefficient
Sen: Sensitivity
Spe: Specificity
Acc: Accuracy
Ppv: Positive predictive value
Npv: Negative predictive value
