# Supplementary material for: Prediction of Ki-67 expression in gastric gastrointestinal stromal tumors using histogram analysis of monochromatic and iodine images derived from spectral CT
Source: Cancer Imaging. 2024 Dec 31;24:173. doi: 10.1186/s40644-024-00820-6 (PMC11686923; doi:10.1186/s40644-024-00820-6)
Supplement: Supplementary file 1 — Supplementary Material 1 [file 40644_2024_820_MOESM1_ESM.docx]

**Table S1.** Inter-observer agreement of histogram parameters

| **Parameters** | **Intraclass correlation coefficient (95% Confidence interval)** | |
| --- | --- | --- |
|  | **Monochromatic images** | **Iodine images** |
| Minimum | 0.960 (0.895, 0.985) | 0.882 (0.714, 0.954) |
| Maximum | 0.966 (0.912, 0.987) | 0.925 (0.810, 0.971) |
| Mean | 0.983 (0.954, 0.993) | 0.990 (0.974, 0.996) |
| Perc.01 | 0.857 (0.658,0.944) | 0.949 (0.870, 0.981) |
| Perc.10 | 0.933 (0.829,0.974) | 0.989 (0.971, 0.996) |
| Perc.25 | 0.959(0.894, 0.984) | 0.950 (0.974, 0.996) |
| Perc.50 | 0.963 (0.904,0.986) | 0.888 (0.726, 0.957） |
| Perc.75 | 0.984 (0.957, 0.994) | 0.985 (0.961, 0.994) |
| Perc.90 | 0.983 (0.956, 0.994) | 0.953 (0.879, 0.982) |
| Perc.99 | 0.959 (0.894, 0.984) | 0.971 (0.925, 0.989) |
| SD | 0.980 (0.948, 0.993) | 0.951 (0.874, 0.981) |
| Variance | 0.993 (0.981, 0.997) | 0.964 (0.906, 0.986) |
| CV | 0.940 (0.846, 0.977) | 0.938 (0.842, 0.976) |
| Skewness | 0.992 (0.980, 0.997) | 0.895 (0.743, 0.960) |
| Kurtosis | 0.989 (0.970, 0.996) | 0.936 (0.837, 0.976) |
| Entropy | 0.902 (0.759, 0.962) | 0.995 (0.988, 0.998) |

**Notes:** SD, standard deviation; CV, coefficient of variation.
